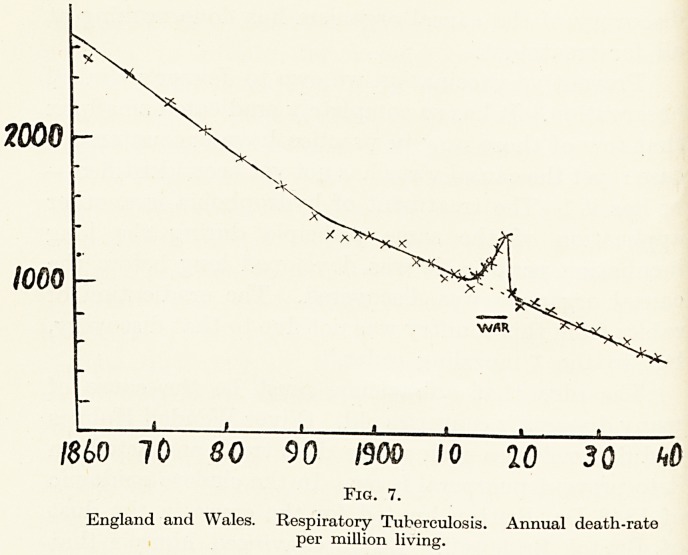# Malum Immedicabile Cancer

**Published:** 1940

**Authors:** E. Watson-Williams

**Affiliations:** Surgeon-in-Charge, Ear, Nose and Throat Department, Bristol Royal Infirmary; Clinical Lecturer in Diseases of the Ear, Nose and Throat, University of Bristol; Laryngologist, Bristol National Radium Centre


### MALUM IMMEDICABILE CANCER.

BY

E. Watson-Williams, M.C., Ch.M.,

*Surgeon-in-Charge, Ear, Nose and Throat Department, Bristol Royal Infirmary; Clinical Lecturer in Diseases of the Ear, Nose and Throat, University of Bristol; Laryngologist, Bristol National Radium Centre.*

### Hard Facts.

In 1936 the population of this country was just over forty millions. There were half a million deaths, of which over 66,000 were due to cancer. Cancer comes second only to heart disease among the principal causes of death, and accounts for ten times as many deaths as do road accidents: since the last Great War it has caused many more deaths than did the war itself.

TABLE I.

Deaths in England and Wales, 1936.*

| Age. | From all Causes. | From Cancer. | Cancer per cent. of "all Causes." |
|---|---|---|---|
| Under 35 .. .. | 104,905 | 1,581 | 1·5 |
| 35—65 .. .. | 142,088 | 30,813 | 21·0 } 16 |
| Over 65 .. .. | 249,771 | 33,960 | 13·5 } 16 |
| Total .. .. | 496,764 | 66,354 | 13·3 |

*Economic Importance.*—If cancer affected only the very old, its importance might be little. Whatever the

* Throughout this paper figures are quoted from *The Registrar-General's Statistical Review*, unless otherwise stated. I apologize for the formidable appearance of the Tables: they show the actual facts.

elderly individual himself might feel about it, whether he died of cancer, pneumonia, or from bombing attacks, need not greatly concern the nation. But in the prime of life, between thirty-five and sixty-five, ability and intellectual powers are at their highest, and family, business and national responsibilities are greatest. Yet it is among those in this "socially valuable" period of life that nearly half all deaths from cancer occur, and these amount to one-fifth of all deaths in that period. If matters continue as they are, of all those who reach the age of thirty-five *one in six is going to die of cancer.*

*Cancer is Increasing.*—This cry has been constant for sixty years and more. Not only are deaths due to cancer increasing, but they are increasing more rapidly than is the total population—the *rate* is increasing.

TABLE II.

ENGLAND AND WALES.

| | | 1891. | 1901. | 1911. | 1921. | 1926. | 1931. | 1936. |
|---|---|---|---|---|---|---|---|---|
| Population, millions .. | | 29·1 | 33·2 | 36·1 | 37·9 | 39·1 | 39·9 | 40·8 |
| **Deaths from Cancer ..** | | — | — | **35,902** | **46,022** | **53,220** | **59,346** | **66,354** |
| Annual Death-rates. | **Cancer, per million living.** | **692** | **842** | **992** | **1,215** | **1,362** | **1,484** | **1,625** |
| | Phthisis, per million living. | 1,599 | 1,264 | 1,062 | 884 | 771 | 742 | 583 |
| | Cancer, plus Phthisis | 2,291 | 2,106 | 2,054 | 2,099 | 2,133 | 2,226 | 2,208 |
| | All causes, per 1,000 living. | 20·2 | 16·9 | 14·6 | 12·1 | 11·6 | 12·3 | 12·1 |

The increase in the cancer death-rate is shown in Table II and as a graph in Figure 3. In the latter, observe that the war had no effect on the real death-rate from cancer, since war casualties affected a younger section of the population; but after 1918, when over 100,000 persons died of influenza (sending the death-rate up to 17·6, instead of the 14·0 expected) there is a definite drop in cancer deaths. Although the brunt of influenza fell on younger persons, it looks as if it killed off enough of those just about to develop cancer to diminish the cancer death-rate for some years.

FIG. 3.

England and Wales. Cancer. Continuous line: Annual death-rate per million living. Broken line: "Standardized" annual death-rate. The distance between the two curves is a measure of changes in composition of the population, trivial before 1900, increasing ever since.

There has been a very considerable increase in the cancer death-rate, even within the last ten years. Is it possible that within so short a time the composition of the population has materially changed? Table IV shows how great this change has been: there is a very large increase in the number of persons who die after fifty-five—that is, of course, in the number who live beyond that age. Indeed, between 1926 and 1936, the increase in deaths after sixty-five is much greater than

TABLE

England and Wales. Deaths from

| Ages. | 35—45 | 45—55 |
|---|---|---|
| 1921 | 35,126 (*3,187=9·1%*) | 44,379 (*8,492=19·1%*) |
| 1926 | 28,511 (*3,138=11·0%*) | 45,629 (*9,042=19·8%*) |
| 1936 | 25,274 (*3,504=13·8%*) | 45,937 (*9,401=20·5%*) |
| Increase 1926–36 | (*366*) | 308 (*359*) |

Figures in brackets are cancer deaths, and the total increase in deaths. Since cancer is almost confined to the second half of life, we might *expect* this "ageing" of the population to be reflected in an increase in cancer deaths. Cabinet Ministers may deplore "an ageing population," but it can only be avoided if the individuals who compose that population have sufficient altruism to die young. In that case it is conceivable that there would be a reduction in deaths from cancer, though there are substantial disadvantages in this method of attaining even so desirable a result.

From Table IV we see that of *all* deaths the proportion due to cancer is steadily increasing: this is obvious also when we remember that for many years the death-rate from "all causes" has been falling, while the cancer death-rate has been rising. But this is not *wholly* due to increase in the proportion of those who live to reach old age. A very serious feature of Table IV is the steady increase in cancer in early middle age. Between 1926 and 1936 the increase in cancer deaths at ages 45—55 was greater than the total increase in deaths from all causes. In the group aged 35—45 there has been a steady and considerable

IV.

All Causes at Different Ages.

| 55—65 | 65—75 | Over 75 | All Ages. |
|---|---|---|---|
| 61,527 | 80,495 | 85,178 | 458,629 *(46,022=10·3%)* |
| 67,450 | 91,497 | 96,528 | 453,804 *(53,220=11·5%)* |
| 82,582 | 119,079 | 130,801 | 496,764 *(66,354=13·3%)* |
| 15,132 | 27,582 | 34,273 | 43,060 *(13,134)* |

percentage due to cancer of all deaths in each group.

*decrease* in deaths from all causes; but cancer deaths are increasing: in 1921 cancer was responsible for 9 per cent. of all deaths in this age-group, in 1926 for 11 per cent. and in 1936 for nearly 14 per cent.

The effect of change in the composition of the population can be eliminated by constructing a table to show, not what actually happens, but what would have happened if the age-group composition of the population had been always what it was in 1901.

The broken line in Figure 3 shows in the form of a graph the changes in the annual death-rate from cancer as they would have appeared if the composition of the population had remained constant. From it we see that during almost a century there has been a steady increase in the "standardized" death-rate from cancer until about fifteen years ago, since when the rate has remained constant.

The distance between the two curves is a measure of changes in the composition of the population, which were very small up to 1900 and have been increasing ever since. The suggestion has been made that after allowing for these changes the remaining increase in cancer is apparent, not real, and due to increased

TA[illegible]

Deaths from Cancer at Va[illegible]

| Age. | Males. Deaths from Cancer. | Males. Millions living. | Males. Death-rate per million. | Fema[illegible] Deaths from Cancer. All sites. | Fema[illegible] Deaths from Cancer. Breast and Uterus. | Milli[illegible] livin[illegible] |
|---|---|---|---|---|---|---|
| Under 25 .. | 304 | 7·86 | 39 | 237 | *10* | 7·7[illegible] |
| 25—35 .. | 454 | 3·32 | 137 | 586 | *203* | 3·4[illegible] |
| 35—45 .. | 1,219 | 2·70 | 412 | 2,285 | *1,118* | 3·1[illegible] |
| 45—55 .. | 3,799 | 2·33 | 1,630 | 5,602 | *2,593* | 2·7[illegible] |
| 55—65 .. | 9,045 | 1·92 | 4,710 | 8,863 | *3,176* | 2·[illegible] |
| 65—75 .. | 11,126 | 1·09 | 10,200 | 10,050 | *2,619* | 1·3[illegible] |
| Over 75 .. | 5,643 | 0·37 | 15,300 | 7,141 | *1,698* | 0·5[illegible] |
| Total over 35 .. | 30,832 | 8·41 | 3,660* | 33,941 | *11,204* | 9·9[illegible] |
| Total .. | 31,590 | 19·59 | — | 34,764 | *11,417* | 21·[illegible] |

* Cancer is negligible for ages under thirty-five, so that the rate calcu[illegible]

accuracy in diagnosis. Can we accept the corollary that in this country as recently as 1880 half of all *deaths* due to cancer—and a third of all *deaths* due to cancer of mouth, skin, breast or œsophagus—were wrongly certified ?

Be that as it may, we must not be hypnotized by an imaginary state of affairs, such as is represented by a "standardized" population. We have to deal with facts—with the population as it is—and must therefore concentrate on the upper curve, and remember that *year by year there is a steady increase in the number, and in the actual rate, of deaths by cancer.*

*Sex and Age.*—In Table V cancer deaths for 1936 have been analysed into groups by sex and age, with cancer of uterus and breast shown separately. One-third of all cancer deaths in women, and one half of cancer deaths under 60, are due to cancer in these sites, where the maximum incidence is much earlier in

GES. ENGLAND AND WALES, 1936.

| Death rate per million. | | Persons. | | | |
|---|---|---|---|---|---|
| All sites. | Omitting Breast and Uterus. | Deaths from Cancer. | Per cent. of all Cancer Deaths. | Millions living. | Death-rate per million. |
| 30 | *30* | 541 | 0·8 | 15·64 | 35 |
| 163 | *110* | 1,040 | 1·6 | 6·81 | 152 |
| 734 | *409* | 3,504 | 5·2 | 5·81 | 603 |
| 2,050 | *1,368* | 9,401 | 14·2 | 5·07 | 1,854 |
| 4,030 | *2,585* | 17,908 | 27·0 | 4·12 | 4,338 |
| 7,500 | *5,546* | 21,176 | 32·0 | 2·43 | 8,715 |
| 12,130 | *9,225* | 12,784 | 19·2 | 0·95 | 13,450 |
| 3,400* | — | 64,773 | 97·6 | 18·396 | 3,520* |
| — | — | 66,354 | — | 40·838 | — |

[illegible] lives above this age gives a truer picture than if all ages are used.

life than for cancer elsewhere in either sex. If we omit cancer of breast and uterus from our calculations we see that for the earlier years the death-rate is almost exactly the same for the two sexes. Figure 5 (p. 16) illustrates the age incidence.

Another point concerning cancer of breast and uterus is that the results of treatment of cancer in these sites are very much better than for cancer anywhere else (Table VI): which means that the difference between cases and deaths, elsewhere inconsiderable, is here quite material. In fact, some two-fifths of all women's cancer occurs in these sites. Although the cancer death-rate for men over thirty-five is higher than for women, the actual case-rate is slightly higher for women than for men. When we note from the table how many more women than men there are in the population at all adult ages we can understand why hospital figures show a very much greater incidence of cancer in *women*, though the Registrar-General's figures give a slightly higher death-rate for *men*.

The third deduction we make from this table is that *the death-rate from cancer is rather more than doubled with the passage of each decade.* Does this not imply an ætiology related to actual "ageing," not merely the passage of time ?

*Sites.*—The distribution of cancer by sites is shown in Table VI, pp. 18-19. For many sites the two sexes are equally affected, but not for all. If we express the number of deaths in females as one, we find the deaths for males are approximately as follows : lip, 12 ; tongue, mouth, tonsil, 8 ; pharynx, œsophagus, larynx,

Skin cancer, both sexes, shown × 10.

FIG. 5.

Deaths from cancer at different ages. England and Wales, 1936. Cancer of testis shows age-incidence like that of uterine cancer.

lung, 3; jaw, rectum, bladder, 2; gall bladder, 0·5. These ratios have changed very little since 1901. But during that interval the increase in cancer has been conspicuous in certain sites. Cancers of lung are *six times* as frequent as before, of prostate about three times, of colon and ovary twice as frequent: on the other hand, cancers of liver, jaw and mesentery are only half as frequent as formerly.

*Status.*—In 1921 it was pointed out that if the male population is arranged in economic grades—well-to-do, artisan, labourer, etc.—the incidence of cancer in the lower grades is materially greater than in the higher. The difference appears chiefly in certain sites: it is greatest for lip, tongue and tonsil, less for larynx, œsophagus and skin, small for stomach (lung was not considered). There is a curious resemblance between this list and that for site-sex-difference: but we might equally claim that the "economic" differences appear in squamous epithelium. A similar survey of married women, classified according to husband's economic grade, shows the same state of affairs for the uterus, the lowest grade having about twice the cancer incidence of the highest: the effect is diminished by a reverse grading for cancer of breast. It has been suggested that the differences are due to variations in standards of cleanliness. For women the effect might be related to frequency of pregnancies; cancer of the uterus is more frequent in the multipara, of the breast in the sterile. Whatever the explanation, it seems clear that rich and plentiful food, wines, and what we may call the material comforts of life, so far from tending to produce cancer, if they have any effect, are beneficial.

The town dweller is as well off as the countryman. For example, the cancer death-rate for ages 55—75 is

TA[illegible]

ENGLAND [illegible]

Deaths recorded in 1935.

| Site. | Males. | Females. | Total. | Per ce[illegible] of Tot[illegible] |
|---|---|---|---|---|
| Tongue, Lips, Jaws, Pharynx .. | 2,972 | 537 | 3,509 | 5·[illegible] |
| Œsophagus .. .. | 1,772 | 705 | 2,477 | 3·[illegible] |
| Stomach .. .. .. | 6,926 | 5,604 | 12,530 | 19·[illegible] |
| Colon and Rectum .. | 5,823 | 5,213 | 11,036 | 17·[illegible] |
| Liver .. .. .. | 1,177 | 1,200 | 2,377 | 3·[illegible] |
| Other Abdominal Organs | 2,936 | 3,649 | 6,585 | 10·[illegible] |
| | 21,606 | 16,908 | 38,514 | 59·[illegible] |
| Larynx and Lung .. | 3,492 | 1,107 | 4,599 | 7·[illegible] |
| Bladder, Prostate, etc. .. | 3,572 | | | 5·[illegible] |
| Bladder, Ovary, etc. .. | — | 2,694 | — | 4·[illegible] |
| Uterus .. .. .. | — | 4,470 | — | 6·[illegible] |
| Breast .. .. .. | 77 | 6,768 | — | 10·[illegible] |
| | | 13,932 | 17,581 | |
| Skin .. .. .. | 606 | 483 | 1,089 | 1·[illegible] |
| Bones, Brain, etc. .. | 1,466 | 1,287 | 2,753 | 4·[illegible] |
| All sites .. .. .. | 30,819 | 33,727 | 64,546 | 100 |
| 1934 .. .. .. | — | — | 63,263 | |

The deaths represent deaths of all persons dying *with* cancer. [illegible] where cancer was regarded as irrelevant, we find that there is a red[illegible] and cancer of skin 150 less (15 per cent.).

slightly higher is rural areas than in London and the large cities. We may shade the map of Great Britain according to local incidence of cancer. The industrial North is perhaps a little darker than the South and East, but the "black spots" are Anglesea and Flintshire!

For many years there has been a close resemblance between the main features and, if we allow for differences in age-group composition of the populations, the incidence of cancer in this country, Australia,

ALES. CANCER.

Cases Estimated for 1934.

| Per cent. survive five years. | Total Cases. | Survive 5 years. Cases. | Survive 5 years. Per cent. of Total Cases. |
|---|---|---|---|
| (1,404) 15 | 1,654 | 250 | 0·36 |
| (2,105) 4 | 2,191 | 86 | 0·12 |
| ? | 2,477 | — | — |
| 4 | 13,051 | 521 | 0·77 |
| 13*a* | 12,663 | 1,627 | 2·23 |
| ? | 2,377 | — | — |
| ? | 6,585 | — | — |
| | 40,998 | — | — |
| ? | 4,699 | (?100) (*b*) | (0·15) |
| 2 | 6,394 | 128 | 0·18 |
| 26*c* | 5,962 | 1,492 | 2·04 |
| 30 | 9,779 | 2,934 | 4·02 |
| (R. 500) 60*d* | 1,250 | 750 | 0·96 |
| (E. 589) 18 | 721 | 132 | 0·18 |
| 2 | 2,808 | 55 | 0·07 |
| — | 72,621(*f*) | 8,075 | 11·08 |
| | *56,880(e)* | *3,549(e)* | *6(e)* |

NOTES.

(*a*) Colon 7 per cent., rectum 18 per cent.: about equal numbers.

(*b*) About 100 patients of all sites marked " ? " guessed to survive.

(*c*) 95 per cent. cervix: body of uterus survival rate 2 per cent., survivors included in the 100 under (*b*).

(*d*) Rodent ulcer about 45 per cent. of total, Epithelioma 55 per cent.

(*e*) As line above, but excluding breast and uterus.

| (*f*) Cases. | Per million over 35. |
|---|---|
| M. 32,806 | 4,025 |
| F. 39,815 | 4,110 |

cept the physician's opinion as to *actual cause* of death, excluding those about 2,500 in all, or 4 per cent. Cancer of breast is 500 less (7 per cent.)

United States, France, Germany, Holland, Denmark, Belgium, Switzerland, etc. When we go farther afield comparisons become difficult. Like a winter's day, primitive or savage life tends to be short, dull and dirty. With flood, famine, dangerous diseases, parasites, reptiles, animals and humans as competitors cancer takes a back place. And in this sense we must agree that cancer is a disease of civilization. But no race—indeed, no species—is exempt.

In Bavaria cancer of the stomach is more frequent than with ourselves, in Holland cancer of the uterus; but the total cancer incidence is not greater. The negro is stated to show a relative immunity: but in the United States, uncorrected for age differences, it is only as three to four compared with whites. India shows a much lower crude cancer mortality than England: but the whole of India and also Bombay exhibit an incidence of cancer, per 1,000 of population at each age, exactly the same as our own. A survey of all in-patients in Indian hospitals shows precisely the same incidence of cancer among the different races and creeds, meat-eaters and vegetarians, wine drinkers and abstainers. In Ceylon betel-nut cancer of the mouth is frequent, in Java cancer of the liver: but neither country shows a total cancer rate higher than India. In Egypt the Mohammedans claim a relative immunity, but this has not been subjected to test by age-groups. Though it is said that Jews have a relative immunity to cancer of penis and uterus, they show in every State a total cancer-rate identical with that of the Gentiles.

*Sarcoma* ought really to be considered apart. In age incidence it shows far less differentiation than carcinoma; it appears sometimes definitely to originate from a single trauma; it has been cured by an attack of erysipelas, spontaneous or artificial; and it is far more prone than carcinoma to dissemination through the blood, instead of through lymph vessels. It affects mainly the kidney, bones, brain and lymphatic tissues. But it forms only a small part, some 3 per cent., of all cancer: and although the conditions governing its appearance and progress may differ from those affecting carcinoma, they seem in all probability to be at least of the same order.

### TREATMENT.

For many years great emphasis has been laid on the importance of early diganosis and the danger of delay. As regards cancer in a few situations it may be true that the results of treatment could be much improved if patients sought advice sooner. But how far is that true of cancer of the lungs, liver, alimentary tract, ovary, uterus and prostate—that is, of three-fourths of the whole ? Early cancer is painless and in a very large proportion of cases symptomless : quite often, indeed, metastases attract attention before the primary growth has been noticed at all. If every surgeon were carefully to analyse his cases, cancerous and innocent, I feel fairly confident that the proportion in which he could point to avoidable delay as a factor *materially* affecting results would be quite small. X-ray examination and the Bendien or similar tests cannot be relied upon to exclude early cancer, and it is clearly impossible to advise every adult with vague or trivial symptoms to submit to laparotomy. In fact, carcinophobia is already causing a whole heap of trouble. I do not believe that with our present equipment we can hope, by reducing the period between first significant symptoms and beginning treatment, to obtain very materially better results.

*Surgery.*—Even at best, surgical treatment of cancer is a somewhat crude business, amounting to nothing less than " amputation " of the affected parts. Man will get along and manage to support existence after the loss of a leg, stomach, colon, organs of generation, or even a lung or a large part of the brain : but it is idle to pretend that this is anything more than making the best of a bad job—mutilation is preferred to an otherwise inevitable, though lingering and painful, death.

*Radiation.*—To avoid a major operation would seem a material gain. Few patients, and not all doctors, understand how severely radiation taxes a patient. The problem is to kill the growth without (quite) killing the patient: the margin is often very small and occasionally overshot. In fact, it is not much use producing more powerful plant until we have bred a more robust race of patients. As it is, many growths are resistant to radiation. By mere chance the first growths to be radiated were rodent ulcers: had epitheliomata been chosen, the history of radiation might well have been different. In comparing results with those of surgery we have to remember that many patients are treated by radiation for whom surgery could offer nothing; and even where the patient does not survive five years there may be considerable gain in comfort as well as survival longer than without treatment.

*Assessment.*—In assessing results of any form of treatment we encounter many difficulties. We will use the word "cure" to mean survival in health and without evidence of recurrence for five years. There will be a few patients who survive five years, but later die of a recurrence: perhaps these will balance those lost sight of and written off as dead who have, nevertheless, really survived (I know from personal experience that this sometimes happens). Merely to secure a complete five-year follow-up is extremely difficult, even in times of peace: war conditions will make it impossible. Another difficulty is to secure a reasonably even standard of selection and classification of cases.

But the real trouble begins when we try to assess the figures we obtain. There are many series giving results of cases treated, but very few stating what proportion of all cases are considered suitable for treatment. The surgeon who refuses all but favourable cases may show a better proportion of cures than one who treats every case not perfectly hopeless : yet the latter may actually have a better ratio of cures to total cases. Good results have a greater probability of being published than poor. And when a surgeon is known to be interested in a particular condition, not only does his increasing experience improve his results, but he tends to see an undue proportion of favourable cases; since professional fame may attract operable cases, but not the hopeless—that is to say, his cases are preselected. There may therefore be some difference of opinion as to the average proportion of cures assigned below to various classes of cancer : I believe, however, that these differences will not prove serious. The basis of the figures given is mainly Short's *Index of Prognosis* and Choyce's *System of Surgery*, 1935.

*Cancer of mouth.*—Tongue : 303 cases, 185 operated on (18 died of operation), 25 per cent. of 185 or 15 per cent. of whole cured. 473 radiated, 32=7 per cent. cured. Choyce says 20 per cent. cured after radium treatment for selected cases. Lip : results much better, but numbers too few to affect figures for group.

*Throat.*—Surgery gives good results for very rare early cancer of vocal cord : elsewhere results poor. Radiation (often with operation as well)=4 per cent. cured. Harmer (*Radiotherapy*, 1932), 1,662 patients, 41 cured.

*Stomach.*—Radiation useless. 528 cases, 156 operated on, 10=2 per cent. cured. Mayo gives 2,094 cases, 736 operated (91 died of operation), 184=9 per cent. cured—much better than any other figures. Average apparently 4 per cent. cured.

*Colon.*—Radiation useless. 60 per cent. suitable for operation (1 in 4 die of operation), 7 per cent. cured. Rectum : 40 per cent. suitable for operation and /or radiation, 18 per cent. cured.

*Breast.*—Early cases, 30 per cent. of total, 80 per cent. cured after operation and radiation (radiation alone gives worse results). Mayo says 70 per cent. early, 22 per cent. late and 36 per cent. of all cases cured. Halsted gives 40 per cent. of all *treated* cured : Leeds 36 per cent. : Guy's (1926) 28 per cent. Average=30 per cent. of all cases cured.

*Uterus.*—Cervix : 3,703 cases, 2,239 operated (mortality 16 per cent.), 23 per cent. cured. Radiation is replacing surgery : 57 per cent. early, 18 per cent. late, 26 per cent. of all cases cured.

*Prostate.*—1,000 cases, 164 operated on, 21 cured—nearly all cases in which growth so small that it was only discovered after operation : radiation less successful. Bladder : 708 cases, 219 treated, 18 per cent. of these cured. For whole group, bladder, prostate, ovary, etc., apparently 2 per cent. cured.

*Skin.*—Nearly half rodent ulcer, radiated 60 per cent. cured. Epithelioma, 18 per cent. cured.

Gullet, liver, other abdominal organs, lungs and mediastinum, bones, connective tissue and brain : An occasional lucky patient is cured.

It is clear that if 26 per cent. of all cases of cancer of the uterus are cured, 1000 deaths represent $1000 \times \frac{100}{74} = 1351$ cases. Assume that all patients who develop cancer in 1934, and are *not* cured, will die in 1935 : a large proportion actually will do so, and we may expect those who survive until 1936 to balance those who developed cancer in 1933 and died in 1935, and so forth.

The left-hand portion of Table VI shows the actual deaths from cancer in 1935, arranged by sites and sex. The right-hand portion is necessarily very speculative. But if we accept the figures given above as approximately correct, we can work out at least roughly how many cases of cancer occurred in 1934 in each site to correspond with the deaths in 1935. The only large source of error is in cancer of the breast. If we take

36 per cent. instead of 30 per cent. for our "cures," the survivors in this group are 3,850, instead of 2,934 : the total survivors are 916 more, that is, 9,456, 12·9 per cent. of total cases : and a small adjustment must be made in all the figures in the last column. As a check, an entirely different set of results was analysed : the "cures" worked out at just under 13 per cent., compared with the 11 per cent. (or perhaps 12·9 per cent.) of the Table. This suggests that the errors are at least not enormous.

If we accept Table VI we see that, corresponding to the 64,546 deaths from cancer in 1935, some 72,000 or 73,000 *cases* of cancer developed in 1934. If the calculated survivals are too few, the number of actual cases was even greater.

But the main features of Table VI must induce a profound melancholy. Can it be that of all those who develop cancer only 11 per cent. survive five years—and if we exclude cancer of the breast and uterus, a mere 6 per cent survive ?

Surely this cannot be true. The efforts that have been made and the progress achieved during the last twenty years must, we feel, have produced better results than these. Is not the truth buried under the possibly large errors of the table ? It may be so : but if in reality we are obtaining better results in treatment, how is it that every year there is a considerable rise in the number of deaths, and in the death-rate from cancer ?

### THE CAUSE OF CANCER.

*Research.*—It seems to be considered axiomatic that to discover the "cause" of cancer will help us to prevent or to treat it : and that the way to do this is by "research," meaning laboratory research. This confounds two different problems, the conditions necessary to initiate the changed cell-behaviour that we call cancer and those involved in maintaining or controlling the process once it has begun. With a simple deficiency disease — myxœdema, beri - beri, anæmia — prevention and treatment may be the same. Otherwise the discovery of the cause of a disease may enable us to *prevent* it in proportion as the cause can be avoided without inconvenience: but only too seldom does such a discovery assist *treatment.*

The discovery of the cause of a disease may enormously assist diagnosis. And laboratory research will often crown with the clarity of experimental demonstration the hard-won deductions of clinical experience. Seventy years ago our grandfathers employed cod-liver oil to cure human babies suffering from rickets—rank "empiricism." Innumerable laboratory analyses failed to show any difference between this substance and olive oil or cream, so that many clinicians took the analyst's word for it and agreed that there was no difference. But now when we use cod-liver oil to cure rickets in baby rats, that is "science."

In the reign of Elizabeth iron was used for anæmia, oranges for scurvy, quinine for malaria and mercury for syphilis. The discovery of the causal organisms of the two last did nothing for treatment: the only material advance in the treatment of G.P.I. was due, not to the discovery of the treponema, but to the purely empirical observation that improvement often followed a fever. In the 'fifties Dr. Budd showed that typhoid was infectious, and also how to avoid it; though it needed the death of the Prince Consort to make us put our "house of office" in order. The discovery of the causal organism has done nothing at all for treatment.

Preventive vaccination we owe to Jenner's clinical observation. It has so completely eradicated smallpox that few of those now in practice have encountered a case : yet the causal virus has not yet been identified— or has it ? The treatment of hydrophobia is another application of the same principle during the long incubation period : it was developed long before the causal organism was discovered. The eradication of rabies from this country was not due to that discovery, but to the "muzzling order."

The idea that animalculæ must be the cause of many diseases is centuries old. Oliver Wendell Holmes and Semmelweiss each assumed its truth and acted on it to prevent puerperal fever. In the cholera epidemic of 1848 Dr. Brittan hunted for the cause in the dust of Bristol Bridewell. Lister convinced himself that "something" introduced from without was the only possible explanation of post-operative septicæmia and had actually begun to practise "antiseptic surgery" before he or anyone had seen the "vibrios." Laboratory investigation immediately confirmed his deductions. He showed us how to *prevent* streptococcal infection ; but the discovery of the causal organisms did nothing to help treatment of the established disease, apart from providing an antiserum : we had to wait seventy years for the sulphonamides.

It is true, of course, that the discovery of causal organisms has given us about a dozen antisera, and preventive vaccination for about the same number of diseases. Beyond this how little has it helped treatment ! Look at Figure 7 in which the death-rate for respiratory tuberculosis is shown in a graph. The straight line demonstrates a uniform rate of decline

FIG. 7.

England and Wales. Respiratory Tuberculosis. Annual death-rate per million living.

during nearly a century, broken only by a sharp rise during the Great War and resumed after that as though it had never been. Perhaps the decline has been a little *less* rapid since about 1900 when sanatorium treatment and tuberculosis clinics were developed. But as far as we can see from this graph neither the teaching that phthisis is infectious, the discovery of the causal organism, the introduction of tuberculin nor, indeed, any subsequent improvement in treatment has had the very slightest beneficial influence on the death-rate.

*Theories of Cancer.*—There appears to be no limit to the number, nebulosity or indeed silliness of opinions put forward. Most of them have this in common that they offer us no help at all in the matter of treatment, nor even as a rule any practicable suggestions for prevention. Even if, for example, the theories of the "back-to-nature" school could be substantiated, reversion to savagery is no more a practicable method for controlling cancer than the "Brave New World" plan of euthanasia for everybody at seventy. Happily the available evidence exonerates all the material blessings of civilization, except perhaps longer life itself, from any part in the causation of cancer. Again, supposing it were shown (against all present evidence) that heredity is important. A woman marries at twenty-five, and at fifty-five, when she is already a grandmother, develops cancer: *que faire?*

The theories of "fœtal rests" and of "tissue-tension" are incapable of demonstration and lead us nowhere. If lymph stasis causes cancer, why do we not see it with elephantiasis? And if intestinal stasis is responsible for this among all other human ills for which it is blamed, can the *Mens conscia recti* school explain why the commonest first symptom of bowel cancer is the onset of constipation previously absent? Two recent books indict respectively the use of gas-fires, and the consumption of fat contrary to the Levitical prohibition. Obviously it is only a matter of time for the blame to be laid on bananas, wireless, contraception or the diminished consumption of beer!

Fortunately we have to treat patients, not diseases. It matters little, therefore, that we have no theory at all—nor even that we have a totally wrong theory, provided we are ready to look at the matter as a whole and do not focus our attention entirely on a single, isolated aspect of the subject.

*Precancerous Conditions.*—Cancer may develop in tissues subjected to long-continued irritation. The irritation may be dermatitis, seborrhœic, arsenical or "varicose-vein"; it may be a broken tooth, syphilitic leukoplakia, a clay pipe, or betel-nut chewing. But we must not make too much of skin cancer. It accounts for only 3 per cent. of all cancer, and differs from all other cancer in that the bulk of the deaths come in extreme old age (Fig. 5): and the skin is obviously subjected to irritations both in kind and degree that are not comparable with conditions affecting other tissues.

Gall-stones are common, often symptomless: few of the patients develop cancer of the gall-bladder. But 80 per cent. of cancerous gall-bladders contain calculi, and it is said that pebbles introduced into the gall-bladder of a guinea-pig lead to cancer. On the other hand, kidneys and bladders containing calculi are not specially prone to malignant disease. Peptic ulceration is common, yet gastric ulcers seldom become malignant and duodenal ulcers practically never. I was taught that irritation from corsets accounted for cancer of the breast: the "explanation" has gone with the corsets.

With all these conditions we feel there is something missing. The incidence of cancer is very low. Can it be that the only importance of the irritation is to determine the *site*, when a cancer is due to develop somewhere or other? Irritation from soot, tar and paraffin, and from X-rays and other rays requires separate consideration: and burns, including the Kangri cancer of Kashmir, should probably be included with these.

In many of the conditions just mentioned papilloma precedes cancer. In the bladder and rectum papillomata are not common, but frequently become malignant: though they account for only a small proportion of cancers in these sites. In Egypt bilharzia is a common cause of papillomatosis, so we may claim it as a "cause" of cancer. With us a rare form of family hereditary papilloma of the rectum almost uniformly goes on to cancer. But since we know nothing of the ætiology of papillomata in this country, the observation that they are frequently precursors of cancer does not help us to the cause of the cancer.

A large proportion of the women with dysphagia-with-anæmia develop cancer of the œsophagus, though usually not in the region where the lesions of the former condition are obvious. But if anæmia is a factor in cancer, why has the disappearance of chlorosis not produced an improvement? Men are subject to anæmia and far more than women to cancer of the œsophagus; but in them there is no apparent relationship, nor do they get dysphagia with their anæmia. There must be some other factor (? endocrine) to account for the relationship in women.

*Tar.*—Chimney-sweeps' cancer has been recognized for sixty years, and mule-spinners' for half as long: but there has been very little change in the mortality from cancer of the scrotum. We realise, however, that soot, tar, and certain oils contain something capable of producing papilloma and even cancer when applied to the skin—that is, of actually initiating the cancerous process. The truth of this has been confirmed by laboratory experiment: and a whole series has been synthesized of new organic bodies, the benz-pyrenes, possessing this carcinogenic power in very high degree. The soot, tar, etc., apparently contain small quantities of these or similar bodies: and this probably also explains the tendency for cancer to develop in the scars of burns.

These discoveries might be of little interest to most of us. We saw however that cancer of the lung has enormously increased since the beginning of this century. Then it was a pathological curiosity, except among miners in the Schneeburg district, where the dust in the mines is radio-active. In 1901 it caused ten deaths per million males, in 1931 fifty-one and in 1936 ninety ! The only suggestion that fits the facts, including occupational liability, points to inhalation of dust from "tarred" roads.* The asphalt and the dust have been shown experimentally to be carcinogenic. If there is anything in this, the sooner we find some other road dressing the better !

*Virus.*—At least once a year we hear of the discovery of the "bacillus" or nowadays the virus of cancer, and the production of a curative serum. Yet there is no evidence at all that cancer is ever transmitted by infection.† The very absence of antibody formation is one of the chief troubles of the clinician. And virus diseases habitually take their toll early in life.

In birds there occur several tumours, of which the Rous sarcoma is the best known, that can be transmitted by means of cell-free extracts, presumably containing a virus. It is claimed that the virus has actually been identified in the ultramicroscope. It is both tissue-specific and species-specific : that is, it will produce only one variety of growth wherever inoculated and is active only in one species of bird. Assume that these tumours are of the same nature as mammalian cancers, though no mammalian virus-cancer is known. A carcinogenic agent will produce cancer in any species of animal, the type depending on the tissue to which it is applied—squamous carcinoma, columnar carcinoma, sarcoma, etc. If therefore a virus is *necessary* to cancer formation, every animal of every species must normally carry as many kinds of virus as there are kinds of cancer : which does not

* Lung cancer has, however, increased also in countries where the roads are not tarred.

† Compare, however, Dr. Markham Skerritt's paper in our first number, in which he demonstrates with copious references that "phthisis is not contagious" : and Culpeper's *Treatise of the Pestilence*, in which he shows that "the pestilence is not infectious."

help much. Even the ingenious theory of Dr. Gye does not avoid this dilemma.

The Shope skin papilloma is readily transmitted, naturally or by a cell-free extract, from one rabbit to another. Such papillomata frequently become malignant, but are then no longer infective. It is unfortunate that so much experimental work on cancer has been done with sarcomata, which as we have observed form a numerically small class that differs in several important respects from the bulk of cancer.

*Hormones.*—In general, cancer shows no or at most a very slight and debatable hereditary tendency. But with cancer of the breast, uterus and prostate there is an incidence considerably above the average in the descendants of patients. By inbreeding we can produce strains of mice with a very high or a very low susceptibility to spontaneous breast cancer. A recent observation has rather upset the apple-cart: it is claimed that if a mouse, for example, of high-cancer strain is suckled to a low-cancer foster-mother it will later on behave in respect to mammary cancer as if it belonged to the foster-mother's strain.

The administration of large doses of œstrogen to female mice (or even to castrated males, normally immune) leads to the development of breast cancer. Indeed, fear of a similar effect can be traced in all papers on the clinical use of œstrogen. From the opposite side, pregnancy seems to activate breast cancer, and oöphorectomy has been employed empirically to control its progress. Add to this that cancer of uterus and breast have an incidence considerably earlier in life than other cancers. Is not the inference that cancer in these sites is related to the activity of the endocrines that normally produce tissue-proliferation and are closely connected with normal growth control: and that perhaps it is an endocrine peculiarity that tends to be hereditary? A comparable observation is that one-third of the patients with Paget's disease die of bone sarcoma: this leads us to the relationship of cancer to the factors controlling calcification.

*Rays.*—Repeated exposure to radium or to X-rays is followed by dermatitis that frequently goes on to cancer. Even the rays of the sun, though apparently innocuous here, are held responsible for a considerable amount of skin cancer in whites in Australia and the tropics. The comparative immunity of the negro to this, and indeed to all cancer has been attributed to the protection afforded by his black skin against sunlight. In the last Long Fox lecture Dr. Chesterman reported that in the Congo the commonest sites for cancer are the palm and sole, which are not protected by pigment.

We know that one important effect of sunlight is the formation of calciferol; which can also be produced by the action of X-rays (on ergosterol). Though there is no experimental evidence that this is carcinogenic, should we question the wisdom of uncontrolled ultra-violet light baths and high-vitamin diet for adults?

Calciferol is essential for normal growth, calcification and the healing of wounds and ulcers. It is exactly this latter property that has established the reputation of cod-liver oil and sunlight in tuberculosis. We are all, it seems, inoculated with tubercle early in life: the development of the disease is evidence of inadequate calciferol. We may use the phthisis death-rate of a community or nation as a measure of the general hygienic standard, that is, healthiness: but more than this, we may use it as a measure of calciferol utilization.

Now there does appear to be a direct antithesis between phthisis and cancer. It is not merely that even in later life phthisis is almost an insurance against cancer. The reason for the relative immunity of the negro to cancer is claimed to be exactly the reason for his liability to phthisis. And in Table II we observed that in this country for the last fifty years the sum of the death-rate from the phthisis and from cancer is constant. In other words, it is suggested that increased healthiness, as measured by the phthisis death-rate, is related to increased cancer not simply because more individuals live into the cancer ages, but directly, the factors that enable us to defeat phthisis actually promoting a tendency to cancer.

*Summary.*—All this would amount to very little if these observations were entirely unrelated. We have the benz-pyrenes, with great power of producing pathological tissue proliferation, and demonstrably carcinogenic. Secondly, œstradiol, normally concerned in tissue proliferation and at least experimentally capable of initiating cancer. And thirdly calciferol, normally necessary for tissue proliferation, and if we accept somewhat oblique evidence perhaps related to carcinogenesis. And *these substances are chemically closely related.*

Whatever view of the origin of cancer we adopt it must agree with the facts: the incidence and general features of cancer are very similar throughout the whole civilized world, and among nations of the same cultural standards as our own practically identical. In this country the townsman is as well off as the countryman, and the well-to-do better off than the poor: so that there is no advantage in an open-air life, hard work or plain or insufficient food. After thirty-five years the incidence of cancer doubles with each decade of age. And all over the world, since as far back as records go there has been a steady increase in the cancer death-rate.

Is it merely by chance, since two-fifths of all women's cancer starts in uterus or breast, that the *total* incidence of cancer in the two sexes is practically identical ? Or that in one country there is a very high incidence of cancer of one organ, in another of cancer of another organ, yet the total incidence is the same ? It seems essential that there is a " general control " : and it is suggested that this is related to excess endocrine activity, possibly perverted, possibly redundant to the later years of life.

We can, if we take the trouble, avoid exposure to soot, tar, oils and X-rays. But this by itself will have no appreciable effect on the bulk of cancer. We cannot expect to prevent cancer if, as is suggested, its origin is bound up with just those factors that render the individual and the race healthy, virile and long-living.

### CONCLUSION.

" Where there is no hope, there can be no endeavour."—*Johnson.*

*Immunity.*—A portion of a cancer may be transplanted, say from one mouse to another, and continue to live and grow as a cancer in the second. Incidentally, after several such transferences the cancer shows a greatly increased power of survival and growth. In this way a cancer may increase to many hundreds of times the size of the whole mouse in which it originated and remain alive and vigorous years after that mouse has perished. But if it is transplanted simultaneously into a number of mice it will not " take " equally in all. In some it grows readily, in one or two perhaps not at all : and perhaps in others after apparently beginning to grow it disappears. We now find that the mice of the third group are very resistant, perhaps completely resistant, to another implantation of the same cancer (and sometimes of other similar cancers): they have developed an immunity. What is more, their serum will confer a certain degree of immunity on other mice, evidence of the production of an antibody.

Unfortunately this method cannot be applied to spontaneous cancer: metastases often grow more rapidly and are more resistant than the primary growth. Whatever antibodies the patient produces they are ineffective. But there is often evidence of *some* antibody formation: (*a*) fibrosis, with destruction of cancer in permeated lymphatics and (*b*) tendency of blood-borne fragments to perish. Only very, very rarely does spontaneous recovery occur: but it has been recorded sufficiently often to place it beyond all doubt.

The occurrence of two or even three entirely separate cancers in the same patient, either simultaneously or successively, has been noted on many occasions. But it is still a rare event, far less common than the "chance expectation." Even when there are numerous papillomata in the rectum, only one becomes malignant. (The case of cancer in both breasts or both tonsils is not under consideration.) Yet there are at any time thousands of persons alive who have had a cancer removed successfully, and who might be expected to have as good a chance as others to develop cancer elsewhere. It appears as if the occurrence of one cancer led to the inhibition of cancer formation, even after the original growth is removed: but there is very little resistance to the original growth if a portion has been overlooked, or even if it is "implanted" accidentally during operation. Though slender, there is *some* evidence of a natural tendency to recovery from cancer.

---

It is manifest, of course, that as matters stand we must continue to encourage patients to seek early treatment: and continue also to develop our present methods of treatment by surgery and radiation. But it does not appear probable that we shall by these means do much to improve the position. The National Radium Commission scolded Bristol for years for its comparative lack of enthusiasm for radium: but it is at least arguable that the local judgment was dispassionate and essentially sound—in only a small field are the results of radium and X-ray treatment anything like as good as the surgeon's.

Nor would I suggest that we should abandon any research. Our national expenditure on this is really ridiculously small. But it should be possible to apply our efforts to greater advantage. We do not require more statistics—we swim already in an ocean of statistics: nor is it easy to discern any advantage in two bodies independently compiling, as now, statistics of the results of treatment of the same set of patients. In the laboratory nearly our whole effort is directed to the (probably sterile) discovery of the cause of cancer: in pharmacology, the field of laboratory research most abundant in fruit for the clinician, nothing is attempted.

Neither as a nation nor as a profession are we sufficiently concerned about cancer. Yet each year the Registrar-General records that the figures of deaths from cancer are the highest ever recorded: "The death-rate for males in 1936 is 109 per cent. higher than in 1901–10." The medical student and the hospital surgeon do not see the whole of cancer. The general practitioner, the Public Assistance officer and the District Nurse know how often death from cancer implies a protracted and hopeless agony.

The difficulty is in great part economic. No surgeon even could afford to confine his work to the operative treatment of cancer. Yet apart altogether from the failures of surgery and radiation there die every year from cancer many thousands for whom these methods offer nothing at all. Working only as a "side-line" and with this unpromising material, a very few optimists, assuming that cancer is a constitutional disease and should be treated by assisting the feeble natural defences, have achieved at least considerable progress. Suppose it were made possible for a few—as a start, even one or two—of those with a wide practical experience in the treatment of cancer patients to devote to these their whole time and attention, unhampered by a pharisaical orthodoxy or *rebus angustis domi*: one cannot help feeling that not only could very much be done to relieve these "miserable objects," but we should bring substantially near the conquest of this grim adversary.

### SUMMARY.

1. Cancer in this country accounts for more deaths each year. Not only is the death-rate increasing, but the rate for each age-group.

2. Nearly half all deaths from cancer occur before sixty-five: cancer accounts for one-fifth of all deaths between thirty-five and sixty-five. Of all who reach thirty-five one in six dies of cancer.

3. The proportion of those who are cured by surgery or radiation is small.

4. Deficiency diseases apart, the discovery of the cause of a disease often does nothing to assist treatment of patients.

5. Cancer can be caused by rays, by bodies of the benz-pyrene group, or by overdoses of œstrogen: these are both chemically related to calciferol, which can be produced by the action of rays.

6. It is suggested that the cause of cancer is constitutional, related to endocrine activity, and integrally connected with factors necessary for health and long life; that we can expect little improvement from education of the public, development of surgery or X-rays or research into the cause of cancer; and that future advances should be sought in pharmacology and in medical treatment.

## Figures and Tables

**Fig. 3. f1:**
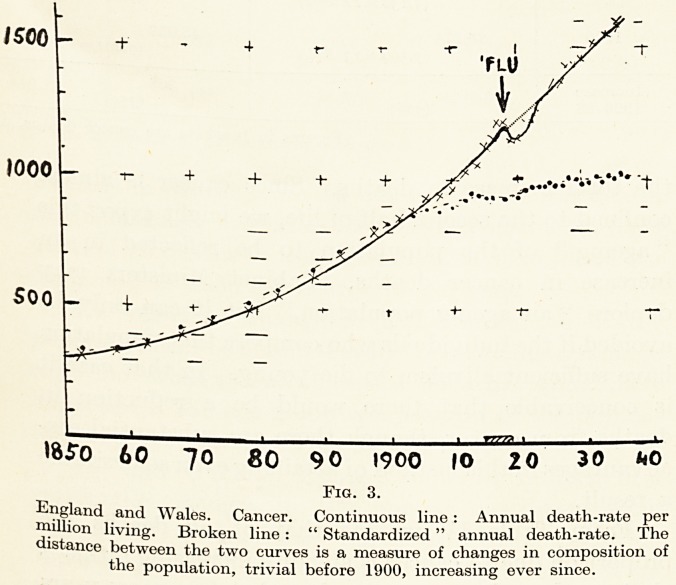


**Fig. 5. f2:**
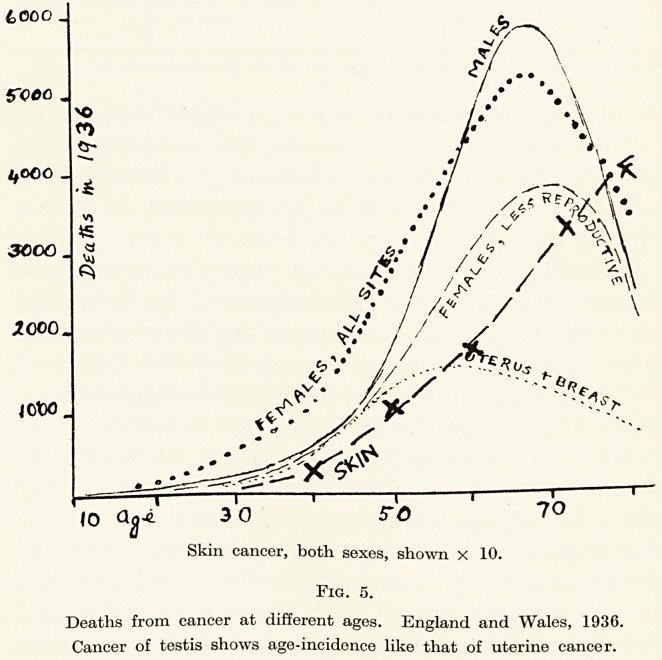


**Fig. 7. f3:**